# Time-Course Gene Expression of ‘*Candidatus* Liberibacter solanacearum’, Prophage, and *Wolbachia* Genes in *Bactericera cockerelli* from Ingestion to *in Planta* Transmission

**DOI:** 10.3390/microorganisms13092120

**Published:** 2025-09-11

**Authors:** Esmaeil Saberi, Jawwad A. Qureshi, Judith K. Brown

**Affiliations:** 1 School of Plant Sciences, The University of Arizona, Tucson, AZ 85721, USA; essaberi323@gmail.com; 2 Department of Entomology and Nematology, IFAS, Southwest Florida Research and Education Center, University of Florida, Immokalee, FL 34142, USA; jawwadq@ufl.edu; 3 Department of Entomology, Michigan State University, East Lansing, MI 48824, USA

**Keywords:** fastidious bacterium, insect vector, psyllid–liberibacter interactions, tomato vein-greening disease *‘Ca*. L. solanacearum’

## Abstract

Psyllids are vectors of fastidious plant pathogenic ‘*Candidatus* Liberibacter’ species that infect both the psyllid vector and plant host. Understanding the molecular and cellular basis of ‘*Ca*. Liberibacter’ interactions with the psyllid host will aid in identification of effectors involved in invasion and multiplication and facilitate transmission to the host plant. The differential expression of previously identified genes/*loci* with predicted involvement in tomato host–plant– ‘*Ca*. L. solanacearum’–prophage–*Wolbachia* endosymbiont dynamics was quantified by RT-qPCR amplification. Fifteen ‘*Ca*. Liberibacter solanacearum genes and/or prophage *loci* and four predicted *Wolbachia* spp. *loci* were analyzed in potato psyllids in a 14-day time-course study, post-48-h acquisition-access period by potato psyllids on ‘*Ca*. L. solanacearum’-infected tomato plants. The ‘*Ca*. L. solanacearum’-infected tomato host plants were used as an infected host ‘calibrator’ species lacking involvement of psyllid effectors. ‘*Ca*. L. solanacearum’ genes with predicted functions in adhesion, motility, transport, and virulence that are associated with the prophage lysogenic lifestyle were differentially expressed. In contrast, the prophage-*loci* expression was synchronous with early or late phase of psyllid-‘*Ca*. L. solanacearum’ infection, respectively. The observations are consistent with the previously in silico-predicted ‘*Ca*. L. solanacearum’ gene and prophage/*Wolbachia loci* functions and time-course global expression patterns. Knockdown of ‘*Ca*. L. solanacearum’ genes involved in invasion, biofilm formation, and colonization would be expected to impair the vertical and horizontal transmission of ‘*Ca*. L. solanacearum’ to psyllid offspring and host plants, respectively.

## 1. Introduction

Several ‘*Candidatus* Liberibacter’ species are pathogens of citrus and solanaceous crop plants that are transmitted by psyllids (Hemiptera: Psyllidae) [1]. Transmission is species-specific, relying on the compatibility between bacterial species, insect vector, and plant host [2]. The two most widespread species, ‘*Ca*. L. asiaticus’ and ‘*Ca*. L. solanacearum’ (haplotypes A and B), are transmitted by the Asian citrus psyllid, *Diaphorina citri* (Kuwayama), and potato psyllid, *Bactericera cockerelli* (Sulc), respectively, in a circulative–propagative manner [1]. The species ‘*Ca*. L. asiaticus’ infects primarily citrus species and is the prevalent and widely distributed pathogen of citrus-greening disease, also known as “huanglongbing” (HLB), a destructive disease associated with fruit drop, yield loss, and eventually decline and tree death [3]. In contrast, ‘*Ca*. L. solanacearum’ infects plant species in the Solanaceae and Apiaceae [4], and is best known for causing zebra chip and vein-greening diseases of potato and tomato plants, respectively [4,5].

Control of vector-borne plant diseases relies heavily on vector management to reduce the population size and thereby lower transmission frequency, which in turn requires an understanding of the biology and dynamics of pathogen acquisition and transmission by the insect vector. Both psyllid age and lifespan are determinants of ‘*Ca*. Liberibacter’ transmission [6,7,8]. Transmission of plant pathogens by the insect vector involves a co-evolved interaction among the insect vector, the pathogen, the plant host, and usually one or more bacterial endosymbionts harbored by the insect vector [9]. Identifying and characterizing the essential genes required to support the ‘*Ca*. Liberibacter’ infection cycle in the plant and insect vector hosts in this pathosystem has been hindered by the inability to establish these fastidious bacteria in pure culture. Further, ‘*Ca*. Liberibacter’ candidate genes predicted to interact with their psyllid and/or plant hosts have been identified primarily through comparative, functional genomic, or proteomic approaches [10]. Genomic analyses have also revealed secretion systems, motility structures, quorum-sensing components, and prophage-like elements [11,12,13,14,15].

In addition to ‘*Ca*. Liberibacter’, psyllids harbor several species of endosymbiotic bacteria, including *Wolbachia* spp. [16,17], which can influence host fitness and potentially affect pathogen interactions [18]. Although psyllid ‘*Ca*. Liberibacter’–*Wolbachia* interactions are not well-studied, *Wolbachia* spp. may contribute to the interplay between the psyllid vector and ‘*Ca*. Liberibacter’ pathogen during the infection cycle. It has been suggested that *Wolbachia* may contribute to vector–pathogen dynamics, possibly by influencing prophage regulation within ‘*Ca*. Liberibacter’ [19]. Finally, comparative transcriptome analysis of ‘*Ca*. L. solanacearum’-infected potato psyllids identified differentially-expressed contigs with similarities to *Wolbachia* proteins with ankyrin repeat motifs, which are known to mediate protein–protein interactions such as signal transducers and transcriptional initiators [20].

While optimal feeding or ingestion parameters required for acquisition and transmission of ‘*Ca*. Liberibacter’ by the psyllid vector have been characterized [6,21], and several psyllid-encoded proteins have been associated with plant-to-plant transmission [20,22], little is known about the molecular and cellular basis of circulative–propagative transmission in these pathosystems. Understanding these mechanisms requires knowledge of both bacterial and psyllid genes, including contributions from bacterial endosymbionts such as *Wolbachia.*

The objective of this study was to investigate the dynamics of previously identified differentially-expressed genes and *loci* encoded by ‘*Ca*. L. solanacearum’ and associated prophages [23] and *Wolbachia* spp., respectively [20], with predicted involvement in interactions between the psyllid vector, ‘*Ca*. Liberibacter’, and *Wolbachia* in this pathosystem. To achieve this, relative transcript expression for selected genes was quantified by real-time quantitative polymerase chain reaction (RT-qPCR) amplification in adult potato psyllids, after a 48-h acquisition-access period (AAP) on ‘*Ca.* L. solanacearum’-infected tomato plants, followed by a 14-day time-course study. The goal was to characterize gene expression during putative bacterial ingestion, multiplication, circulation, and potential salivary gland acquisition to better understand the molecular interactions that facilitate CLso transmission in the potato psyllid–tomato pathosystem.

## 2. Materials and Methods

### 2.1. Potato Psyllid Insect Colony Establishment and Maintenance

‘*Ca*. L. solanacearum’-infected and -uninfected potato psyllids were collected in 2012 from infested tomato plants growing in a commercial greenhouse in Arizona and maintained on ‘Roma’ tomato *Solanum lycopersicum* Mill. in insect-free growth chambers [20]. Colonies were reared in separate rooms at 24 ± 2 °C, 50% relative humidity (RH), and 14:10 (L:D) photoperiod (LED lights). The presence or absence of ‘*Ca*. L. solanacearum’ in the potato psyllid colonies was determined by PCR amplification of the 16S rRNA gene using the previously published primers OA2/OI2c [24] with monthly testing of 25 adults from each colony. The ‘*Ca*. L. solanacearum’ isolate was identified as haplotype A using a single-nucleotide repeat (SSR) marker (primers SR-1F and Lso-SSR-1r) [25]. Psyllid cohorts were identified as ‘Central’ type based on a mtCOI phylogenetic marker [26].

### 2.2. Infection Rate, Transmission Efficiency, and ‘Ca. L. solanacearum’ Accumulation, or Load, in Infected Potato Psyllid Colonies

Laboratory colonies of *‘Ca*. L. solanacearum’-infected adult potato psyllids were tested monthly to track *‘Ca*. L. solanacearum’ concentration in relation to seasonal variation and potential fluctuation associated with colony health or rearing conditions. Each month, four biological replicates, each with 10 individual teneral adults, were analyzed for bacterial concentration (load) by qPCR analysis. Transmission efficiency was assessed using tomato seedlings (2-leaf stage) exposed to teneral adult psyllids reared on ‘*Ca*. L. solanacearum’-infected tomato plants. Individual psyllids were transferred gently to tomato test plants with a hand-held aspirator and confined in cup cages (Figure S1) at 25 °C, 50% RH, and a 16:8 (L:D) photoperiod for a 14-day inoculation-access period (IAP). Psyllids were collected on day 7, frozen at −80 °C, and stored for DNA extraction; leaf samples were collected from the inoculated tomato plants at 14 days post-IAP.

Total DNA from psyllids and plants was extracted using the cetyltrimethylammonium bromide (CTAB) method [27] and stored at −80 °C until analysis. The ‘*Ca*. L. solanacearum’ 16S rRNA gene (rDNA) was amplified by qPCR amplification using the primers and probe LsoF-qPCR-F/-16S-qPCR-P/HLBr-qPCR-R (Tables S1 and S2). The standard curves were prepared from the serial dilutions (10^1^–10^10^ copies) of a cloned 16S rDNA fragment in DNA from uninfected psyllids or plants [28]. Genome copy number was calculated from DNA mass using standard molecular weight conversion [29]. Each reaction contained 10 ng total DNA for psyllid samples or 200 ng for plant samples, or the plasmid vector as the negative control. Three biological replicates were each analyzed in triplicate.

### 2.3. Tomato Source Plants for ‘Ca. L. solanacearum’ Acquisition-Access Period

Tomato source plants were established by inoculating 8–10 leaf stage (~14-day-old) tomato plants with 25–30 adult potato psyllids from a colony maintained on ‘*Ca*. L. solanacearum’-infected tomato plants. Psyllids were confined to a single leaf on a middle-tier branch using clip cages [29] for a 14-day IAP. After inoculation, the adult psyllids, eggs, and developing offspring were removed from the inoculated plants by excising the infested leaf with a razor blade. Plants were maintained in psyllid-free cages in a greenhouse at 24 ± 2 °C, 50% RH, and a 16:8 (L:D) photoperiod. Three weeks post-IAP, plants were tested for ‘*Ca*. L. solanacearum’ by PCR and confirmed by qPCR amplification to quantify bacterial accumulation. Symptomatic qPCR-positive plants were subsequently used as source plants for time-course transmission experiments.

### 2.4. Expression of ‘Ca. L. solanacearum’, Prophage, and Wolbachia Genes in Potato Psyllids at Different Inoculation-Access Periods Following a 48-h Acquisition-Access Period on Tomato

Expression of ‘*Ca*. L. solanacearum’-prophage and *Wolbachia* spp. genes (Table 1) was quantified over a 14-day time course, at 2-day intervals during a two-week IAP on tomato test plants, following a 48-h AAP on ‘*Ca*. L. solanacearum’-infected tomato source plants.

For the IAP study, approximately 300 ‘*Ca*. L. solanacearum’-free teneral psyllid adults were transferred to either infected or uninfected tomato source plants. After 4 h, individual psyllids were transferred to ‘*Ca*. L. solanacearum’-free tomato test plants (2–3 leaf stage), each confined in a 160 mL clear plastic cup cage (mesh top for ventilation; Figure S1) under controlled conditions (24 ± 2 °C, 50% RH, 14:10 L:D photoperiod). Adult psyllids were allowed an inoculation-access period (IAP) of 0, 2, 4, 6, 8, 10, 12, or 14 days on tomato test plants. At each time point, ten psyllids were collected from each test plant and pooled for RNA extraction, and five psyllids were collected and pooled for DNA extraction. At the completion of the 14-day IAP, the newest expanding leaves (100 mg) were collected from each tomato test plant. Total DNA was extracted from pooled psyllid and tomato leaf samples using the CTAB method and subjected to RT-qPCR amplification as described above. The genome copy number, corresponding to ‘*Ca*. L. solanacearum’ concentration in both the psyllid and plant hosts, was calculated per above.

### 2.5. Selection of Genes and Loci for Reverse Transcription Quantitative PCR (RT-qPCR) Amplification

Nineteen genes or *loci* of interest, consisting of 15 ‘*Ca*. L. solanacearum’-prophage genes/*loci* and four endosymbiont *Wolbachia* spp. genes, were selected for RT-qPCR amplification (Table 1). Selection of the targets (Table 1) of interest was based on previous results [20,23] and additional in silico analyses of laboratory transcriptome databases (Dr. Judith K. Brown Lab, School of Plant Sciences, University of Arizona, Tucson, AZ, USA) to identify new or previously predicted genes associated with the ‘*Ca*. L. solanacearum’ circulative–propagative pathway or other relevant ‘*Ca*. L. solanacearum’-potato psyllid interactions.

‘*Ca*. L. solanacearum’ orthologs were identified based on a homology search using a protein query against *‘Ca*. L. asiaticus’ strain psy62 (NC_012985.3) and UF506 (HQ377374.1) reference sequences, and the ‘*Ca*. L. solanacearum’-NZ genome reference (NC_014774.1; A haplotype). Searches were performed using tblastn and blastx, with E-value cutoffs of ≤0.00 and ≤1 × 10^−5^, respectively. The top 10 BLAST hits for each sequence were functionally annotated using NCBI GenBank (https://www.ncbi.nlm.nih.gov/genbank; accessed May 2023), UniProtKB-Swiss-Prot (https://www.uniprot.org; accessed May 2023), and NCBI Conserved Domain databases (https://www.ncbi.nlm.nih.gov/Structure/cdd/cdd.shtml; accessed May 2023). Conserved protein domains were predicted using InterProScan (https://www.ebi.ac.uk/interpro/interproscan.html; accessed May 2023), and sequence motifs were identified using PROSITE Scan (https://prosite.expasy.org/scanprosite; accessed May 2023). The coding sequence with the highest BLAST (https://blast.ncbi.nlm.nih.gov/Blast.cgi; accessed May 2023) similarity score and coverage, and the most biologically relevant annotation(s), was confirmed/verified by amino acid homology searches of the corresponding bacterial chromosomal gene or prophage *locus*, respectively.

The predicted ‘*Ca*. L. solanacearum’ orthologs for *‘Ca*. L. asiaticus’-associated prophages SC1 and SC2 (HQ377374.1) [30] were identified using viroBlast [31] and PHAST (PHAge Search Tool) [32] algorithms, using the default parameters for homology and prophage region searches, respectively. The orthologs were selected based on the best match among the ten top BLAST hits (viroBlast) with the highest similarity score and coverage with the predicted prophage region of the ‘*Ca*. L. solanacearum’-NZ genome reference (NC_014774.1), hereafter, “predicted prophage genes” to distinguish them from ‘*Ca*. L. solanacearum’ or *Wolbachia* spp. chromosomally-encoded orthologs. Finally, five predicted endosymbiont *Wolbachia loci* from the potato psyllid transcriptome (J.K. Brown Lab, School of Plant Sciences, University of Arizona, Tucson, AZ, USA) were annotated using the gene annotation pipeline as described above, and genes of interest were selected for expression analysis [20].

Primers for the predicted ‘*Ca*. L. solanacearum’ and *Wolbachia* spp. orthologs were designed based on genome sequence of ‘*Ca*. L. solanacearum’-NZ in NCBI (NC_014774.1) and the potato psyllid transcriptome (unpublished, Dr. J. K. Brown Lab, School of Plant Sciences, University of Arizona, Tucson, AZ, USA), respectively (Tables S1 and S2).

### 2.6. Polymerase Chain Reaction (PCR) and Reverse Transcriptase PCR Amplification, Cloning, and Sequencing

Total DNA was extracted from teneral adult stage psyllids using the CTAB method. Primers (Table S1) were designed using the IDT Primer Quest Primer Design Tool. Reactions were carried out in a 20 μL reaction mixture containing 2 μL of template DNA, 10 μL 2× JumpStart REDTaq ReadyMix PCR Reaction Mix (Sigma, St. Louis, MO, USA), and 40 nM of each primer, in an Eppendorf Master Cycler Gradient Thermocycler (Hamburg, Germany). Cycling parameters consisted of an initial denaturation of 4 min at 94 °C, 35 cycles of 20 s denaturation at 94 °C, 30 s annealing at 55–60 °C (Table S1), 30–90 s extension at 72 °C, and final extension of 10 min at 72 °C. Amplicons were separated by agarose gel (1%) electrophoresis in 1 × Tris-Acetate-EDTA (TAE) buffer, pH 8.0.

Amplicons were cloned into the pGEM-T Easy plasmid vector (Promega, Madison, WI, USA) and transformed into chemically competent *Escherichia coli* DH5α cells. Positive colonies were identified by colony PCR amplification [33], and inserts were verified by bi-directional Sanger DNA sequencing (Eton Biosciences, Inc., San Diego, CA, USA) using M13 primers. Consensus sequences, assembled from three or more reads, were confirmed by BLAST against ‘*Ca*. L. solanacearum’/‘*Ca*. L. asiaticus’ or *Wolbachia* spp. references. Plasmids were purified with a GeneJET Plasmid Miniprep Kit (Thermo Scientific, Waltham, MA, USA) and quantified using a NanoDrop spectrophotometer (ND-1000, NanoDrop Technologies, Wilmington, DE, USA).

### 2.7. Extraction of RNA, cDNA Synthesis, and Reverse Transcriptase PCR

Total RNA was extracted from the adult potato psyllid using Tri Reagent (Invitrogen, Carlsbad, CA, USA) and homogenized in a bead beater (BioSpec Products, Bartlesville, OK, USA) with 0.5 mm zirconium beads (RPI Research Products, International, Mount Prospect, IL, USA). RNA extraction followed the manufacturer’s protocol, and residual DNA contamination was removed using the DNA-free™ DNA Removal Kit (Invitrogen, USA). cDNA was synthesized from the DNase-treated RNA using the High-Capacity cDNA Reverse Transcription Kit (Applied Biosystems, Carlsbad, CA, USA). For total RNA isolation from ‘*Ca*. L. solanacearum’-infected tomato plants, 100 mg of newly expanding leaves were macerated and homogenized with 3–3.2 mm stainless steel beads in TRIzol^®^ Reagent and processed as above.

### 2.8. Reference Gene Selection for RT-qPCR amplification of ‘Ca. L. solanacearum’-Prophage and Wolbachia Genes/Loci

The concentration of ‘*Ca*. L. solanacearum’ cells was expected to vary depending on the host (e.g., tomato plant vs. potato psyllid), number of days post-inoculation, or relative age of potato psyllids used for time-course experiments. To select a suitable reference gene for the gene expression studies, the correlation coefficient between the expression level of the reference genes 16S rRNA [34], *recA* [35] and *gyrB* [19] (Table 1), and ‘*Ca*. L. solanacearum’ genome copy number, was evaluated based on the cycle quantification (Cq) value. Data were considered at all treatments for ‘*Ca*. L. solanacearum’-infected tomato plants and adult potato psyllids at each time-course interval. The correlation coefficient between ‘*Ca*. L. solanacearum’ concentration (genome copy number) and gene expression were 0.96 ± 1.85, 0.92 ± 6.32, and 0.91 ± 6.52 for *recA*, *16srRNA*, and *gyrB*, respectively. The strongest correlation was observed for *recA* (r > 0.96), indicating it was the most stable reference gene. Accordingly, *recA* was selected as the suitable ‘*Ca*. L. solanacearum’ reference gene for expression studies.

Expression of the *Wolbachia* genes/*locus* was normalized using a *Wolbachia* spp. *FtsZ* as the reference gene [36]. To determine if *FtsZ* was an informative reference gene for RT-qPCR analysis, expression of *FtsZ* was compared to *Wolbachia* gDNA Ct values for both’*Ca*. L. solanacearum’-free and ‘*Ca*. L. solanacearum’-infected potato psyllids at each post-IAP interval in this study. A strong Pearson correlation coefficient of r > 0.92 was found between *FtsZ* and *Wolbachia* gDNA Ct values. Based on these results, *FtsZ* was selected as the reference gene for normalizing W*olbachia* target gene expression.

### 2.9. Quantitative Polymerase Chain Reaction Amplification

Quantitative PCR assays were carried out using the CFX96 Touch Real-Time PCR Detection System (Bio-Rad, Lunteren, The Netherlands) according to the manufacturer’s recommendations. Three technical replicates per biological replicate (*n* = 3) were analyzed by RT-qPCR amplification of samples, and a no-template and no-reverse transcriptase control. Primers and probes for ‘*Ca*. L. solanacearum’, prophage, and *Wolbachia* spp. were designed using the IDT Assay Selection Tool, available at the IDT website (Table S2). The reaction efficiency for primer/probe combinations was determined by constructing a standard curve for a 10-fold serial dilution (10^1^ to 10^9^ copies/μL) of cloned target gene fragments.

The reactions (20 μL volume) were carried out in a 96-well plate using amplicon-specific TaqMan probe and primer combinations. Each reaction contained 10 μL of 2xTaqMan Universal Master Mix (Applied Biosystems), 1 μL 20x Primetime primer/probe stock, 5 μL of RNase-free water, and 4 μL of cDNA template. The qPCR cycling parameters consisted of 50 °C for 2 min initially, 95 °C for 10 min, and 40 cycles of a 2-step program (95 °C for 15 s, 60 °C for 60 s).

The relative fold change in expression for each gene of interest was calculated using the delta–delta quantification cycle method (ΔΔCq) [37]. The relative expression results for each target gene were expressed as a ratio of target gene expression to reference gene expression results for each sample and compared to calibrator (normalization) samples. ‘*Ca*. L. solanacearum’-infected tomato plant and ‘*Ca*. L. solanacearum’-free psyllid samples were used as reference samples for ‘*Ca*. L. solanacearum’-prophage and *Wolbachia* gene expression, respectively.

### 2.10. Statistical Analysis

Data normality was analyzed using the Shapiro–Wilk test [38]. For data that followed a normal distribution (*p*-value > 0.05), a parametric approach was used for the analysis. A one-way ANOVA was carried out to detect potential treatment effects, followed by the Fisher Least Significant Difference (LSD) test for mean separation when the ANOVA results were significant (*p* < 0.05), using InfoStat^®^ version 2020. The mean differences between the expression of a gene of interest and ‘*Ca*. L. solanacearum’ accumulation at different adult psyllid time-course intervals were analyzed by one-way ANOVA (*p* = 0.05). Statistical significance (*p* < 0.05) between normalized expression of each gene of interest, relative to expression of the analogous genes/*loci* for the calibrator (normalization) samples, was evaluated using the Student’s *t*-test (*p* = 0.05).

## 3. Results

### 3.1. Transmission Efficiency and Infection Rate of ‘Ca. L. solanacearum’-Infected Potato Psyllid

Infection and transmission efficiency of laboratory ‘*Ca*. L. solanacearum’-infected adult potato psyllids ranged between 90–100% and 23–80%, respectively, during the experiment.

**Figure 1:**
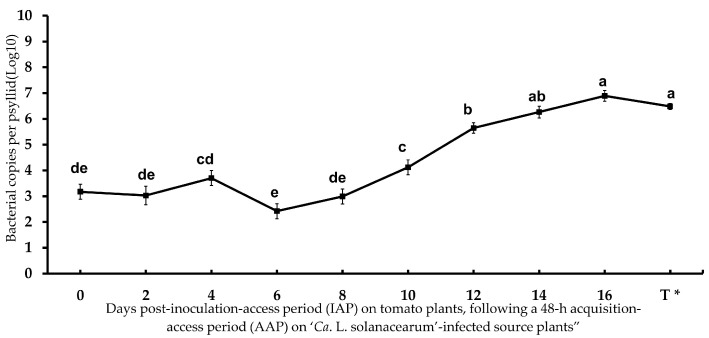
‘*Candidatus* Liberibacter solanacearum’ copy number in adult potato psyllid at different inoculation-access periods (IAP) time points, following a 48-h acquisition-access period (AAP) on ‘*Ca*. L. solanacearum’-infected tomato plants. Error bars represent the standard error of the mean. Different letters indicate a statistically significant difference in bacterial accumulation in adult psyllids among time intervals (ANOVA with Fisher’s LSD test, *p* < 0.05). T * = ‘*Ca*. L. solanacearum’ accumulation in teneral adult potato psyllids that were born and reared on infected tomato plants, used as the infected psyllid source colony in this study.

Psyllid adults tested positive for ‘*Ca*. L. solanacearum’ at a rate of 100% by PCR and qPCR amplification following a 48 h AAP on the tomato source plants, and the bacterium was consistently detected in adult potato psyllids at all time points post-IAP after their transfer to tomato test plants (Figure 1). Statistical analysis showed that the ‘*Ca*. L. solanacearum’ genome copy number in potato psyllids increased significantly after 8 days on tomato test plants (10, 12, 14, and 16 days post-IAP), following a 48-h AAP on infected tomato source plants. By day 14 post-IAP, bacterial accumulation reached similar levels to those in teneral adults that were reared continuously on infected tomato plants. This was consistent with the ‘*Ca*. L. solanacearum’ genome copy number that ranged from 1.8 × 10^3^ to 2.1 × 10^4^ and 2.6 × 10^6^ at 0, 10, and 14 days post-IAP, respectively (Figure 1), indicating a gradual increase in ‘*Ca*. L. solanacearum’ load over time.

The efficiency of ‘*Ca*. L. solanacearum’ transmission based on percentage transmission to tomato plants by teneral psyllid adults was determined with a 48-h ‘*Ca*. L. solanacearum’ AAP on ‘*Ca*. L. solanacearum’-infected tomato plants, and 14-day IAP on ‘*Ca*. L. solanacearum’-free tomato test plants. The results showed that ‘*Ca*. L. solanacearum’ was undetectable in the ‘*Ca*. L. solanacearum’-inoculated tomato plants up to 12 days post-IAP (i.e., 14 days after exposure to the ‘*Ca*. L. solanacearum’ source plant), based on the qPCR amplification results (Table 2). The percentage of tomato plants that tested positive for ‘*Ca*. L. solanacearum’ increased over time, with transmission rates of 13.3, 26.6, and 30.0% at 12, 14, and 16 days post-IAP, respectively (Table 2). The bacterial accumulation (load) ranged from 10^1^ to 10^2^ in infected tomato plants.

### 3.2. Gene Expression of ‘Ca. L. solanacearum’ Predicted Orthologs Encoding Putative Bacterial Flagella and Pili Components

**Figure 2:**
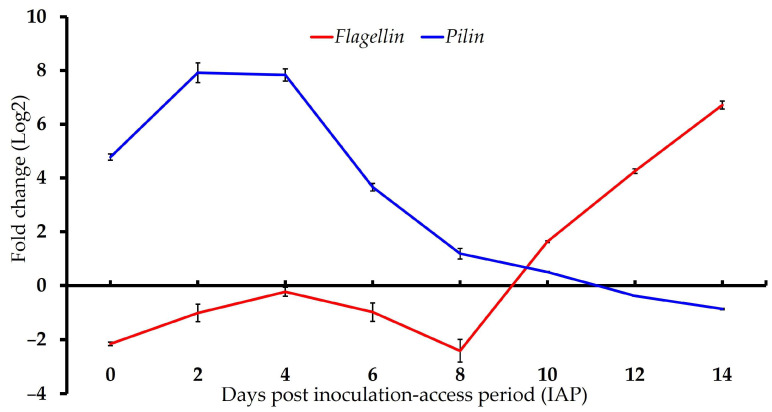
Relative normalized expression of ‘*Candidatus* Liberibacter solanacearum’ genes associated with ‘*Ca*. L. solanacearum’ motility, adhesion/attachment, and propagation/virulence in adult tomato psyllids.

The *loci* associated with the predicted ‘*Ca*. L. solanacearum’ *pili* gene (*AZCH8*) showed increased expression during the early IAP time points, peaking at 4 days post-IAP in adults with a 233-fold increase relative to the expression of the same bacterial gene in the tomato control plants (t(2) = −37.16, *p* = 0.001). Conversely, expression of the predicted flagellum gene (*AZCH6*) decreased through day 8, then increased sharply at later time points, reaching approximately 107-fold by day 14 post-IAP (t(2) = 30.32, *p* = 0.0011) (Figure 2, Table 3).

### 3.3. Gene Expression of ‘Ca. L. solanacearum’ Predicted Orthologs Encoding Putative Virulence Effectors

**Figure 3:**
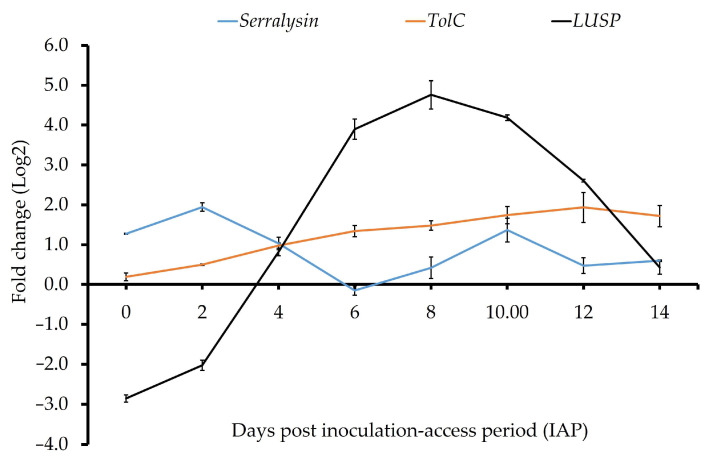
Relative normalized expression of ‘*Ca*. L. solanacearum’ predicted orthologs encoding putative virulence effectors in adult tomato psyllids.

Expression of predicted ‘*Ca*. L. solanacearum’ virulence effector *loci luxR* (*AZch2*) and *imelysin* (*AZch5*) was undetectable at early IAP time points but increased significantly after day 10 post-IAP (Figure 2, Table 3). By day 14 post-IAP, *luxR* expression reached approximately 20-fold (t(2) = 165.81, *p* = 0.001), and *imelysin* reached ~8-fold (t(2) = 112.40, *p* = 0.001). The ‘*Ca*. L. solanacearum’ ortholog *locus* encoding the putative TolC protein (*AZch9*) showed higher expression at all time points in potato psyllid adults compared to tomato calibrator plants with expression increasing over time. The ortholog encoding putative *serralysin* (*AZCH4*) showed fluctuating expression (increased and decreased) across the time course (Table 3), peaking at early (day 2, 5.3-fold, t(2) = 11.38, *p* = 0.007) and late (day 10, 2.4-fold, t(2) = 7.95, *p* = 0.015) time points. The expression pattern of the predicted homolog of the ‘*Ca*. Liberibacter’ genus-specific secretion protein (LUSP) [11] varied over time, with expression increasing after day 4 and peaking at day 8 post-IAP (27.6-fold, t(2) = 23.28, *p* = 0.001; Figure 3, Table 3).

### 3.4. Predicted ‘Ca. L. solanacearum’ Ortholog of Prophage Genes Associated with Phage Cycle Regulation

Predicted prophage-related genes, including *DNA polymerase A* (*AZph17*), anti-repressor (*AZph15*), *integrase/recombinase* (*AZph6*), and repressor protein C2 (*AZph11*), showed a consistent pattern of decreased expression in adult potato psyllids compared to tomato plants over the study time points (Table 3).

**Figure 4:**
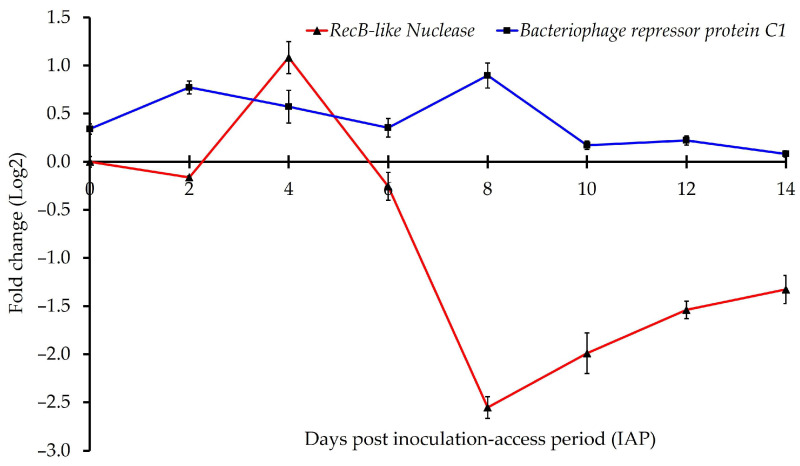
Normalized relative expression of ‘*Candidatus* Liberibacter solanacearum’ genes associated with the ‘*Ca*. L. solanacearum’ prophage infection cycle in adult psyllids.

In contrast, the chromosomal gene encoding the repressor protein C1 (*AZch1*) exhibited significantly higher expression in psyllid adults at the early time points (days 0–8 post-IAP) compared to tomato control plants, with no significant difference observed between the later time points (10-, 12-, and 14-days post-IAP) (Figure 4, Table 3).

The predicted *recB-like nuclease* (*AZph13*) involved in recombination repair [39] showed variable expression across time points, with significant overexpression at day 4 post-IAP (t(2) = 11.0, *p*= 0.008), followed by decreased expression after day 6 post-IAP. Expression of *AZph13* was not significantly different between potato psyllid adults and the control tomato plants from 0- and 6-days post-IAP (Figure 4, Table 3).

### 3.5. Expression of Predicted Phage Genes Associated with Psyllid–‘Ca. L. solanacearum’ Interactions

**Figure 5:**
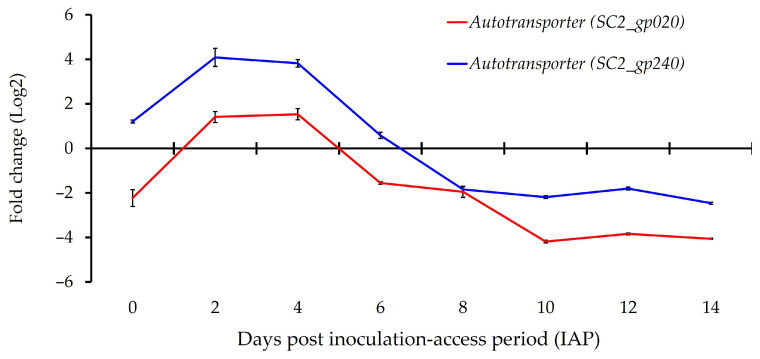
Relative normalized expression of ‘*Candidatus* Liberibacter solanacearum’ *loci* encoding putative autotransporter in adult tomato psyllids during inoculation-access period (IAPs) following a 48-h acquisition-access period (AAP) on infected tomato plants.

Expression patterns of the predicted ‘*Ca*. L. solanacearum’ homolog encoding putative ‘*Ca*. L. asiaticus’ autotransporter adhesion LasAI (*AZph20*) and SC2-gp240 (AZph19) showed variability across time points. Both *loci* reached peak expression between day 2 and day 4 post-IAP, after which expression declined (Figure 5, Table 3). The *AZph19 locus* was over-expressed in potato psyllid adults during the early time points (days 0, 2, 4, and 6 post-IAP) but under-expressed at the later time points, compared to calibrator tomato plants (Figure 4 and Table 3). Similarly, increased expression of *AZph20* was observed at the early time points on day 2 (t(2) = 14.6, *p* = 0.004) and day 4 (t(2) = 17.2, *p* = 0.003) by 2.1- and 3.0-fold, respectively, compared to tomato calibrator plants, and thereafter declined (Figure 5, Table 3).

### 3.6. Expression of Predicted Potato Psyllid-Associated Wolbachia Genes/Loci

Expression of four predicted *Wolbachia loci* was analyzed in ‘*Ca*. L. solanacearum’-infected and uninfected potato psyllid adults across eight time points.

The *locus* encoding putative J domain-containing protein (*AZWo1*) showed significantly higher expression at day 0 (t(2) = −61.7, *p* < 0.001), day 12 (t(2) = −61.7, *p* = 0.011), and day 14 post-IAP (t(2) = 5.4, *p* < 0.032) in infected psyllids, compared to ‘*Ca*. L. solanacearum’-free potato psyllid adults at the same time points.

**Figure 6:**
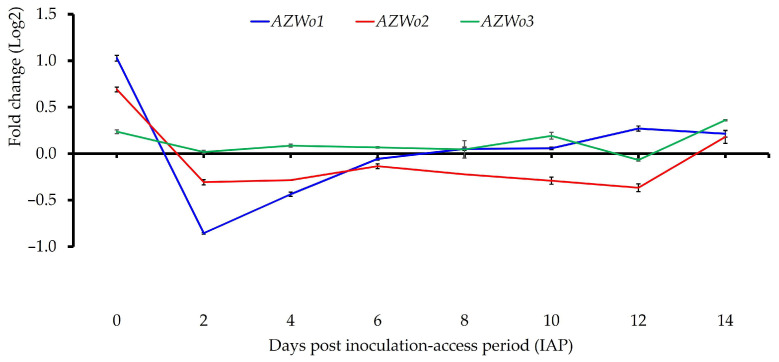
Relative normalized expression of *Wolbachia loci* encoding putative ankyrin repeat-containing proteins in adult tomato psyllids across 0–14 days of the inoculation-access period (IAP), after a 48-h acquisition-access period (AAP).

Two *loci* encoding putative phosphocholine transferase (*AZWo2* and *AZWo3*) showed similar expression patterns, with increased expression at early and late time points 0 (t(2) = 13.2, *p* = 0.006) and 14 days post-IAP (t(2) = 62.3, *p* < 0.001), respectively (Figure 6, Table 3).

Here, expression patterns of the predicted *Wolbachia* repressor protein *locus* (AZWo4), orthologous to the *D. citri*, WDIAC_RS0101550, were evaluated across time-course intervals post-48 h AAP. In potato psyllids, AZWo4 exhibited overall over-expression post-IAP, while significant increases were observed at several time points. A peak was observed on day 12 when expression reached 3-fold higher than controls (t(2) = 10.2, *p* = 0.009). At day 0 of the teneral adult stage, no difference in expression of the *Wolbachia* repressor (AZWo4) ortholog was observed in psyllids compared to the control (t(2) = 0.056, *p* = 0.95) (Table 3).

## 4. Discussion

Teneral potato psyllid adults efficiently acquired ‘*Ca*. L. solanacearum’ within 48 h, but transmission to tomato plants remained low at 12 days post-IAP, consistent with previous reports of high acquisition but moderate transmission rates (~30%) [3,40,41]. Successful transmission required a threshold bacterial load of ~1 × 10^6^ per psyllid and at least 12 days IAP, likely reflecting the latent period needed for bacterial accumulation and salivary gland invasion [3,28]. Teneral adults exposed to bacteria at later stages showed lower transmission efficiencies of 13–30%, whereas the psyllid laboratory colonies that had been continuously reared on infected plants exhibited a higher transmission rate (~62%). The increased efficiency in colony-reared psyllids was likely due to increased bacterial accumulation and a prolonged acquisition-access feeding time [28,40].

Predicted ‘*Ca*. L. solanacearum’ *pili* and *flagellin* genes showed converse expression patterns, with higher relative expression of *pili* during early IAP (0–6 days) and higher expression of *flagellin* during late IAP (8–14 days). This pattern is consistent with previous studies in psyllids, where *pilin* genes are highly expressed during initial gut colonization (day 2–8 after acquisition) [42], while flagellar genes show elevated expression later in the infection cycle and are expressed in psyllids but not in plants [42,43,44]. The results suggest that *pili* and *flagella* play roles in adhesion, circulation through the hemolymph, and movement to the salivary glands [3,28,45], with approximately eight days of IAP required for the bacteria to cross the gut barrier and initiate motility.

Similar expression patterns were observed for *luxR*, *Imelysin*, and *flagellin loci*, with higher expression at later time points (>10 days IAP). Along with increasing ‘*Ca*. L. solanacearum’ genome accumulation, this pattern suggests that the quorum-sensing system [46] may regulate key physiological processes, including the production of secreted proteins, Imelysin, and motility. This coordinated gene expression likely facilitates bacterial propagation and movement within the psyllid hemolymph. Supporting this, transcriptome studies showed significantly higher expression of the ‘*Ca*. L. asiaticus’ *imelysin* gene (*CLIBASIA_02610*, peptidase) in *D. citri* compared to the citrus host (log2FC = 3.5; 11.1-fold change) [44]. Like many bacterial pathogens, liberibacter likely uses Imelysin-like proteins to regulate iron retrieval from the psyllid host, supporting multiplication and systemic infection [47].

The differential expression pattern of the ‘*Ca*. L. solanacearum’ *loci* encoding putative *TolC* (*AZch9*), the third component of the T1SS I secretion system (TISS) [12,13], and putative *serralysin* (*AZCH4*), a potential Type I secretion system (T1SS) substrate [11], suggests their involvement in ‘*Ca*. L. solanacearum’ pathogenicity within potato psyllids. The protease activity of *serralysin* has been proposed as a liberibacter virulence factor that may help evade host antimicrobial defenses [35,48]. The high expression of *serralysin* in the ‘*Ca*. L. solanacearum’ supports this, indicating a potential immune-suppressive role during infection.

The ‘*Ca*. Liberibacter’ genus-specific secretion proteins (LUSP) exhibit temporally regulated expression, with notable upregulation during mid to late stages of the psyllid infection cycle. LUSP, characterized by a signal peptide unique to the genus, is likely under rapid evolutionary pressure, reflecting its specialized role in ‘*Ca*. Liberibacter’–psyllid interactions [11]. In ‘*Ca*. L. solanacearum’, which contains only a single copy of this protein [11], its elevated expression during the infection cycle suggests a critical function in bacterial adaptation within the potato psyllid host.

Overall, the expression analysis of selected prophage-related genes in ‘*Ca*. L. solanacearum’ revealed distinct regulatory patterns associated with lysogeny in the psyllid host and potential lytic activity in the tomato host. Interestingly, the data showed increased expression of a prophage-encoded repressor ortholog, decreased expression of a putative *phage antirepressor*, and differential expression of a *recB-like nuclease* (*AZph13*) implicated in recombination repair. Together, these findings support the hypothesis that a repressor–anti-repressor regulatory system may influence prophage activity in ‘*Ca*. L. solanacearum’, similar to mechanisms reported in other bacterial systems [49]. These results are consistent with prior studies suggesting that lysogeny predominates in the psyllid host, while lytic cycling is negligible or undetectable [30,42,50,51,52]. Although a *Wolbachia*-encoded protein has been proposed to repress the lytic cycle of ‘*Ca*. Liberibacter’ prophages [19], this hypothesis has been debated [49]. The regulatory balance observed here suggests that prophage gene expression may play a central role in maintaining lysogeny and restricting lytic induction in the psyllid host, although the molecular details remain unresolved. Notably, available ‘*Ca*. L. solanacearum’ genomes [13,53] appear to lack a gene ortholog encoding Holin, based on protein and nucleotide BLAST analyses.

These results highlight the elevated expression of ‘*Ca*. L. solanacearum’–predicted autotransporters in the psyllid host. A similar expression pattern has been reported for SC2_gp020 during the early stages of *D. citri* gut colonization [42]. The consistent upregulation of these autotransporters during the early infection cycle suggests they play a key role in the initial stages of liberibacter colonization and adaptation within the insect host. Autotransporters are widely implicated in bacterial virulence and transport processes [30,54], and in other systems, they contribute to auto-aggregation, adhesion, and biofilm formation [55,56]. Such functions may be particularly relevant for facilitating liberibacter colonization and persistence in the insect gut. Together, these observations support the idea that autotransporters may act as an alternative secretion pathway to T3SS or T4SS, providing liberibacter with a versatile strategy for host interaction during early infection [42].

All three predicted *Wolbachia* genes encoding ankyrin repeat-containing proteins—*AZWo1*, *AZWo2*, and *AZWo3*—exhibited similar expression patterns, with increased expression at both the earliest and latest time points examined in ‘*Ca*. L. solanacearum’-infected potato psyllids. These results are consistent with a prior transcriptome study by Fisher et al. [20], which also reported higher expression of *Wolbachia* ankyrin repeat genes in infected compared to uninfected psyllids. Ankyrin repeat genes are widely distributed in insect-associated *Wolbachia* genomes and mediate diverse host interactions, including cytoskeletal dynamics and intracellular trafficking [57,58]. Domain analysis of *AZWo1* revealed a eukaryotic-like DNAJ domain (PF00226) and predicted Type IV secretion system (T4SS) effector features, consistent with reports that ankyrin repeats are often present in bacterial T4SS effectors [59]. Such domains can function as virulence effectors by modulating host vesicular trafficking [59]. In addition, *AZWo2* and *AZWo3* shared homology with *Wolbachia AnkX*, an effector previously shown to disrupt host endocytic recycling through phosphocholination [60]. Disruption of endocytic recycling is a strategy also employed by other bacterial pathogens, including liberibacter, to enhance intracellular survival [60,61,62,63].

Notably, psyllids lacking *Wolbachia* have been reported to transmit ‘*Ca*. L. solanacearum’ less efficiently than *Wolbachia*-infected haplotypes [64]. Together, these findings and the expression data suggest a potential role for *Wolbachia* in shaping liberibacter–psyllid interactions, although the underlying mechanisms remain unclear and warrant further investigation.

## 5. Conclusions

This study investigated the expression dynamics of 19 predicted genes potentially involved in bacterial invasion, multiplication, systemic infection, circulation, and acquisition during an IAP time-course study of ‘*Ca*. L. solanacearum’ infection in adult potato psyllids. The infection process requires the bacterium to overcome multiple barriers in the vector, including the gut, hemolymph, and salivary glands. These results indicate that *‘Ca*. L. solanacearum’ may employ diverse strategies at distinct infection stages, including adhesion, intracellular survival, motility, and immune evasion, to establish systemic infection and reach the salivary glands.

In contrast to ‘*Ca*. L. asiaticus’, infection of adult psyllids by ‘*Ca*. L. solanacearum’ occurred relatively rapidly, with detectable colonization by two days post-acquisition and a short latency period (≤14 days) before transmission. Early colonization (2–5 days) appeared to involve bacterial adhesins, prophage-derived factors (e.g., autotransporters), and potential contributions from *Wolbachia*-encoded ankyrins that may facilitate crossing of epithelial barriers. Progression into the hemolymph (8–10 days) coincided with expression of *flagellin*, consistent with bacterial motility and supported by prior ultrastructural observations. A subsequent rise in bacterial accumulation (load) suggested rapid proliferation, potentially facilitated by the secretion of virulence proteins (Serralysin, Imelysin, and *‘Ca*. Liberibacter’ genus-specific secretion protein) regulated by quorum sensing.

Although tissue-specific gene expression could not be resolved here, the overall patterns suggest that ‘*Ca*. L. solanacearum’ coordinates multiple gene sets in a stage-specific manner to adapt to changing environments and persist within the psyllid. The potential role of prophage repressors in maintaining lysogeny, thereby preventing premature lysis, also emerges as an important area for further study. Moreover, *Wolbachia* effector gene expression highlights possible interactions between bacterial symbionts and ‘*Ca*. L. solanacearum’ that may influence persistence and transmission.

Taken together, the expression profiles summarized here provide a working model of the infection cycle of ‘*Ca*. Liberibacter’ in psyllids, offering candidate molecular targets for future functional studies. These findings contribute to a broader understanding of vector–pathogen interactions and may help identify intervention strategies to reduce psyllid-mediated transmission to plants.

## Figures and Tables

**Table 1:** List of genes (*loci*) selected for expression analysis in potato psyllid adults in this study.

**1-‘*Candidatus* Liberibacter solanacearum’ Chromosomal, Prophage Genes (*Loci*)**			
***Locus* ID ^a^**	**‘*Ca*. L. asiaticus’ Homolog ^b^**	**Homolog ^c^**	**Gene (*Locus*) Annotation**
*AZch1*	*CLIBASIA_01645*	*DJ66_RS00660*	*bacteriophage repressor protein C1*
*AZch2*	*CLIBASIA_02905*	*DJ66_RS03455*	*LuxR* transcriptional regulator
*AZch4*	*CLIBASIA_01345*	*DJ66_RS04950*	*serralysin*
*AZch5*	*CLIBASIA_02610*	*DJ66_RS04795*	*imelysin* domain protein
*AZch6*	*CLIBASIA_02090*	*DJ66_RS00075*	*flagellin*; *FliC*
*AZch8*	*CLIBASIA_03095*	*DJ66_RS03635*	Major fimbrial protein; *Flp1*
*AZch9*	*CLIBASIA_04145*	*DJ66_RS02195*	*TolC*; transporter
*AZch10*	*CLIBASIA_04540*	*DJ66_RS00995*	‘*Ca*. Liberibacter’-genus-specific-protein
*AZch15*	*CLIBASIA_RS00350*	*DJ66_RS05900*	*recA*
*AZch16*	*CLIBASIA_RS03560*	*DJ66_RS00260*	*16S rRNA*
*AZch17*	*CLIBASIA_03525*	*DJ66_RS04070*	*gyrB*; DNA gyrase subunit B
*AZph6*	*SC2_gp065*	*DJ66_RS00645*	*Integrase/recombinase*
*AZph11*	*SC2_gp125*	*DJ66_RS01515*	Phage-related repressor protein C2
*AZph13*	*SC1/2_gp195*	*DJ66_RS05295*	*recB-like nuclease*
*AZph15*	*SC1/2_gp200*	*DJ66_RS00565*	Putative *phage antirepressor*
*AZph17*	*SC1/2_gp210*	*DJ66_RS05280*	*DNA polymerase A*
*AZph19*	*SC2_gp240*	Unknown	Autotransporter adhesion gene
*AZph20*	*SC2_gp020*	*DJ66_RS05330*	Autotransporter; cell wall associated biofilm
**2-*Wolbachia* endosymbiont of *Bactericera cockerelli* genes (*loci*)**			
***Locus*** **ID ^d^**	**SeqID**	**Wo_ DC ^e^**	**Gene/*locus* annotation**
*AZWo1*	BcGS_20518	*HGO53_00485*	J domain-containing protein (*DnaJ*)
*AZWo2*	BcAN_16274	*FK497_06755*	Phosphocholine transferase (*AnkX*)
*AZWo3*	BcAN_05576	*FK497_06755*	Phosphocholine transferase (*AnkX*)
*AZWo5*	BcGS_23202	*FK497_04725*	Core conserved bacterial protein (*FtsZ*)
*AZWo6*	No accession No. ^f^	*WDIAC_RS0101550*	*Wolbachia* repressor protein ^g^

^a^ *Loci* analyzed for ‘*Ca*. L. solanacearum’ haplotype A were designated as the “AZph” and “AZch” for predicted prophage and chromosomal genes, respectively. ^b^ Selected *loci* of ‘*Ca*. L. asiaticus’ strain UF506 (HQ377374.1) and psy62 (NC_012985.3), respectively. ^c^ Predicted homolog of ‘*Ca*. L. solanacearum’ haplotype A (NZ1; NZ_JMTK01000001-5.1) of selected ‘*Ca*. L. asiaticus’ genes for gene expression study. ^d^ *Loci* analyzed for the *Wolbachia* endosymbiont of potato psyllid, *B. cockerelli* ‘Central haplotype’, are designated “AZWo” for selected *Wolbachia loci*. ^e^ Predicted homolog of *Wolbachia* endosymbiont from *D. citri* (strain wDi; CP051608.1). ^f^ Predicted *Wolbachia* repressor-like protein (WP_017531870), hypothesized to regulate the ‘*Ca*. L. asiaticus’ prophage infection cycle in *D. citri.* ^g^ No complete genome reference sequence available for the *Wolbachia* endosymbiont of potato psyllid (Central haplotype); transcriptome sequence reads were identified through an ortholog search of the potato psyllid transcriptome (J.K. Brown Lab, School of Plant Sciences, University of Arizona, Tucson, AZ, USA).

**Table 2:** Transmission of ‘*Candidatus* Liberibacter solanacearum’ by individual adult potato psyllids given a range of inoculation-access periods (IAP) on tomato test plants following a 48-h ‘*Ca*. L. solanacearum’-acquisition-access period (AAP) on ‘*Ca*. L. solanacearum’-infected tomato source plants.

IAP (Day) ^1^	Transmission Rate% ^2^	‘*Ca*. L. solanacearum’ Copy No. (Log10) ^3^
2	0.00% (0/30)	3.03
4	0.00% (0/30)	3.70
6	0.00% (0/30)	2.42
8	0.0% (0/30)	2.99
10	0.0% (0/30)	4.12
12	13.3% (4/30)	5.65
14	26.6% (8/30)	6.26
16	30.0% (9/30)	6.89

^1^ Inoculation-access period (IAP) days of potato psyllid adults on tomato test plants, post-48-h AAP on ‘*Ca*. L. solanacearum’-infected tomato source plants. ^2^ Mean percentage of ‘*Ca*. L. solanacearum’-positive tomato test plants exposed to adult potato psyllids given different IAPs, post-48-h AAP on infected tomato source plants. ^3^ Mean ‘*Ca*. L. solanacearum’ copy number detected for individual adult potato psyllids given different IAPs on tomato test plants, post-48-h AAP on infected tomato source plants.

**Table 3:** Relative normalized expression of genes in tomato psyllid adults after a 48-h acquisition-access period (AAP) followed by 0–14 days of inoculation-access period (IAP).

*Locus* ID (Gene/*Locus* Name) ^1^	Fold Change (Log2 Ratio ± SE) ^2^							
	Inoculation-Access Period Days After 48-h Acquisition-Access Period							
	0	2	4	6	8	10	12	14
*AZch1* (Repressor protein C1)	0.34 ± 0.07 b	0.77 ± 0.12 cd	0.57 ± 0.21 c	0.36 ± 0.10 b	0.90 ± 0.17 d	0.17 ± 0.11 ab ^ϕ^	0.22 ± 0.05 ab ^ϕ^	0.08 ± 0.07 a ^ϕ^
*AZch2* (*LuxR* transcriptional regulator)	ND	ND	ND	ND	ND	0.81 ± 0.2 a	1.78 ± 0.45 b	4.31 ± 0.05 c
*AZch4* (*Serralysin*)	1.27 ± 0.01 cd	1.94 ± 0.11 e	1.03 ± 0.16 c	−0.15 ± 0.12 a ^ϕ^	0.42 ± 0.27 b ^ϕ^	1.37 ± 0.30 d	0.47 ± 0.20 b ^ϕ^	0.60 ± 0.02 b
*AZch5 (Imelysin domain protein)*	ND	ND	ND	ND	ND	−0.18 ± 0.07 a ^ϕ^	0.64 ± 0.06 b	3.07 ± 0.04 c
*AZch6* (*Flagellin*; *FliC*)	−2.16 ± 0.12 a	−1.01 ± 0.65 b	−0.23 ± 0.31 c	−0.98 ± 0.35 b	−2.41 ± 0.66 a	1.63 ± 0.04 d	4.26 ± 0.08 e	6.71 ± 0.38 f
*AZch8* (Major fimbrial protein; Flp3)	4.78 ± 0.19 f	7.91 ± 0.43 g	7.83 ± 0.37 g	3.66 ± 0.28 e	1.18 ± 0.31 d	0.5 ± 0.05 c	−0.38 ± 0.05 b	−0.87 ± 0.05 a
*AZch9* (*TolC*; transporter)	0.19 ± 0.09 a	0.50 ± 0.02 a	0.98 ± 0.07 b	1.34 ± 0.14 bc	1.48 ± 0.12 c	1.74 ± 0.22 cd	1.93 ± 0.38 d	1.71 ± 0.27 cd
*AZch10* (‘*Ca*. Liberibacter’ specific protein)	−2.85 ± 0.09 a	−2.03 ± 0.13 bc	0.82 ± 0.09 d	3.90 ± 0.25 f	4.76 ± 0.35 f	4.18 ± 0.07 g	2.61 ± 0.04 e	0.43 ± 0.17 c ^ϕ^
*AZph6* (*Integrase/recombinase*)	ND	ND	ND	ND	ND	−4.99 ± 0.14 a	−3.98 ± 0.18 b	−2.85 ± 0.11 c
*AZph11* (phage-repressor protein C2)	−1.88 ± 0.18 c	−0.93 ± 0.39 d	−1.78 ± 0.44 c	−3.01 ± 0.21 b	−3.00 ± 0.41 b	−3.67 ± 0.02 a	−3.06 ± 0.06 b	−3.34 ± 0.02 ab
*AZph13* (*recB-like Nuclease*)	0.00 ± 0.09 a ^ϕ^	−0.16 ± 0.02 a	1.08 ± 0.17 e	−0.26 ± 0.15 a ^ϕ^	−2.55 ± 0.24 a	−1.99 ± 0.30 b	−1.54 ± 0.22 c	−1.33 ± 0.27 c
*AZph15* (Putative *phage antirepressor*)	ND	ND	ND	ND	−3.9 ± 0.34 c	−6.33 ± 0.02 a	−5.96 ± 0.05 b	−6.25 ± 0.03 ab
*AZph17* (*DNA polymerase A*)	−2.85 ± 0.30 c	−2.63 ± 0.31 c	−3.00 ± 0.49 c	−4.08 ± 0.05 b	−4.65 ± 0.45 b	−7.29 ± 0.10 a	−7.55 ± 0.84 a	−7.21 ± 0.01 a
*AZph19* (Putative autotransporter)	1.21 ± 0.11 d	4.08 ± 0.48 e	3.82 ± 0.39 e	0.58 ± 0.36 c	−1.84 ± 0.23 b	−2.19 ± 0.17 ab	−1.8 ± 0.07 b	−2.46 ± 0.11 a
*AZph20* (Putative autotransporter)	−2.23 ± 0.97 b	1.41 ± 0.34 c	1.53 ± 0.51 c	−1.55 ± 0.12 b	−1.95 ± 0.64 b	−4.19 ± 0.15 a	−3.84 ± 0.08 a	−4.06 ± 0.04 a
*AZWo1* (J domain-containing protein)	1.03 ± 0.05 e	−0.86 ± 02 a	−0.44 ± 0.04 b	−0.06 ± 0.02 b	0.05 ± 0.03 c ^ϕ^	0.06 ± 0.02 c ^ϕ^	0.27 ± 0.05 d	0.23 ± 0.08 cd ^ϕ^
*AZWo2* (Phosphocholine transferase)	0.69 ± 0.04 c	−0.3±0.05 a	−0.29±0.00 a	−0.13±0.05 a	−0.13±0.05 a	−0.22±0.07 a	−0.37±0.04 a	0.21±0.04 b
*AZWo3* (Phosphocholine transferase)	0.24 ± 0.03 c	0.02 ± 0.03 b ^ϕ^	0.09 ± 0.03 b ^ϕ^	0.07 ± 0.02 ab ^ϕ^	0.05 ± 0.16 ab ^ϕ^	0.19 ± 0.06 c ^ϕ^	−0.07 ± 0.02 a ^ϕ^	0.36 ± 0.01 d
*AZWo6* (*Wolbachia* repressor protein)	0.01 ± 0.12 b ^ϕ^	0.63 ± 0.04 c	−0.58 ± 0.02 a	0.73 ± 0.01 cd	0.81 ± 0.05 cde	0.89 ± 0.03 de	1.65 ± 0.16 e	1.03 ± 0.08 f

^1^ *Loci* analyzed for ‘*Candidatus* Liberibacter solanacearum’ haplotype A, designated “AZph #” and “AZch #” for the prophage and chromosomal genes, respectively. The loci of the *Wolbachia* spp. endosymbiont associated with the potato psyllid, *Bactericera cockerelli* AZ ‘Central haplotype,’ are designated “AZWo.” ^2^ The fold change (log2 ratio) represents relative expression of selected genes. For ‘*Ca*. L. solanacearum’ *loci*, expression in infected psyllids was compared to bacterial expression in infected tomato plants. For *Wolbachia loci*, expression in infected psyllids was compared to *Wolbachia* expression in ‘*Ca*. L. solanacearum’–free psyllids. Data were normalized to the chromosomal reference genes *recA* (‘*Ca*. L. solanacearum’) and *ftsZ* (*Wolbachia*). Letters (a–g) indicate statistically significant differences among means at different IAP time points, and are based on Fisher’s LSD test (*p* < 0.05) using InfoStat^®^ version 2020. ^ϕ^ = No significant difference from the control (Student’s *t*-test, *p* > 0.05). ND = no transcript detected (no measurable Cq value) using RT-qPCR amplification in potato psyllid adult samples.

## Data Availability

The original contributions presented in this study are included in the article and Supplementary Materials. Further inquiries can be directed to the corresponding author.

## Supplementary Materials

The following supporting information can be downloaded at: https://www.mdpi.com/article/10.3390/microorganisms13092120/s1, Table S1: Information regarding the PCR primers used in this study. Table S2: List of primers and probes used in this study for real-time quantitative PCR. Figure S1. Cup cage used to confine psyllids on tomato test plants during a 14-day inoculation-access period (IAP), following a 48-h acquisition-access period (AAP) on ‘*Ca*. L. solanacearum’-infected source plants. The cage consisted of a 160 mL clear plastic cup (15 cm height) with the bottom removed and replaced with a mesh fabric top for ventilation.
