# Supplementary figures and images for: Time-Course Gene Expression of ‘Candidatus Liberibacter solanacearum’, Prophage, and Wolbachia Genes in Bactericera cockerelli from Ingestion to in Planta Transmission
Source: Microorganisms. 2025 Sep 11;13(9):2120. doi: 10.3390/microorganisms13092120 (PMC12472774; doi:10.3390/microorganisms13092120)

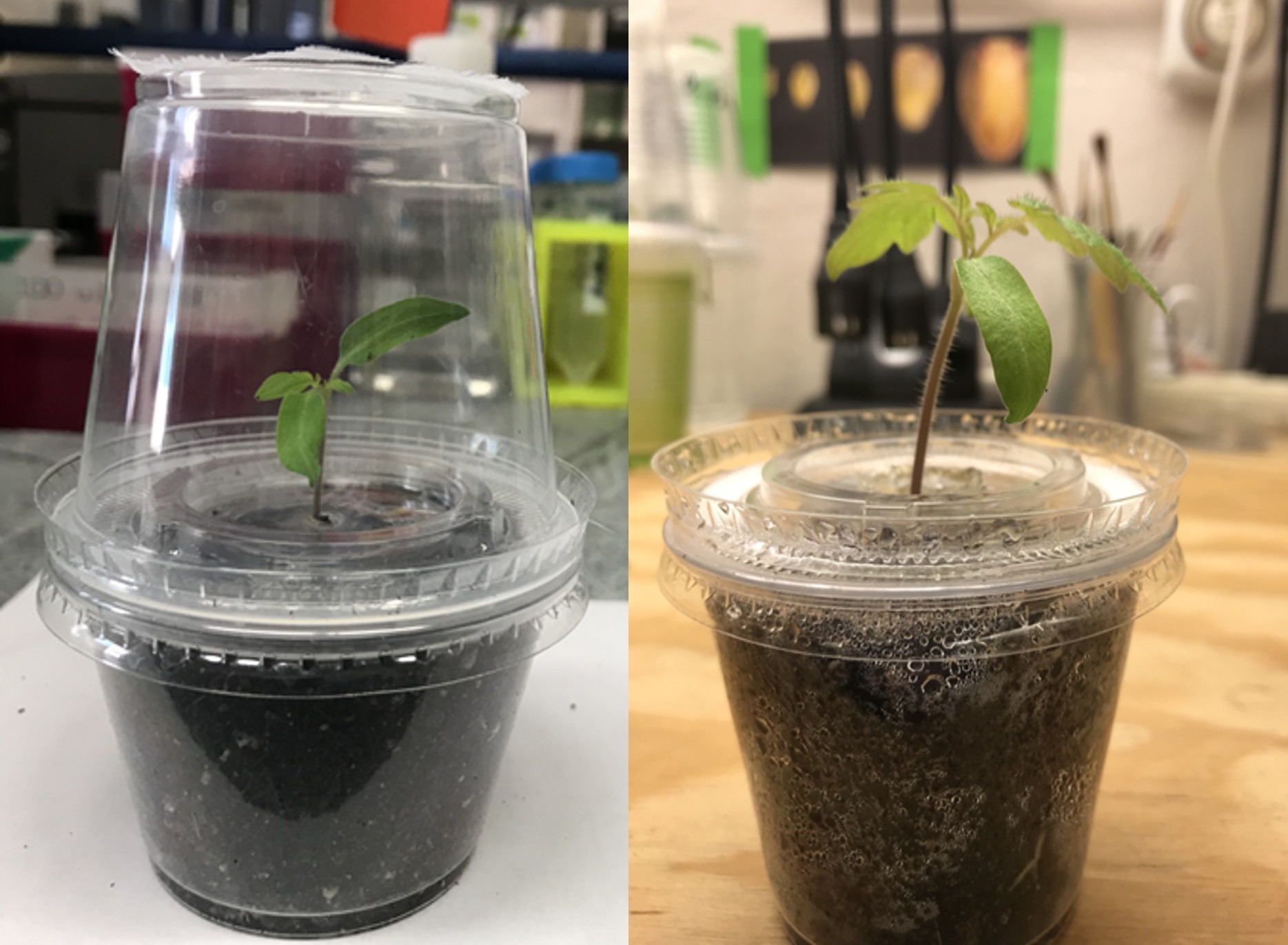

Supplement: Supplementary file 1 [file microorganisms-13-02120-s001.zip › microorganisms-3776307_Supplementary Files/Fig. S1.jpg]
